# Systematic mapping of rare genetic disease studies using UK primary care electronic health records

**DOI:** 10.1038/s41431-026-02114-w

**Published:** 2026-05-20

**Authors:** Thomas E. B. Wright, Hannah Slevin, Sinéad Magnier, Matthew J. Carr, Shruti Garg, Roger T. Webb, Darren M. Ashcroft, Siddharth Banka

**Affiliations:** 1 Division of Psychology and Mental Health, School of Health Sciences, Faculty of Biology, Medicine, and Health, University of Manchester, Manchester, UK; 2 Manchester Centre for Genomic Medicine, St Mary’s Hospital, Manchester University NHS Foundation Trust, Manchester, UK; 3 National Institute for Health and Care Research Manchester Biomedical Research Centre, Manchester University NHS Foundation Trust and University of Manchester, Manchester, UK; 4 Division of Cancer Sciences, School of Medical Sciences, Faculty of Biology, Medicine and Health, University of Manchester, Manchester, UK; 5 National Institute for Health and Care Research Greater Manchester Patient Safety Research Collaboration, University of Manchester, Manchester, UK; 6 Division of Pharmacy and Optometry, School of Health Sciences, Faculty of Biology, Medicine and Health, University of Manchester, Manchester, UK; 7 Child and Adolescent Mental Health Services, Royal Manchester Children’s Hospital, Manchester University NHS Foundation Trust, Manchester, UK; 8 Division of Evolution, Infection and Genomics, School of Biological Sciences, Faculty of Biology, Medicine and Health, University of Manchester, Manchester, UK

**Keywords:** Disease genetics, Epidemiology, Health services

## Abstract

Rare disease studies often rely on small, selected cohorts, are resource-intensive and difficult to scale. UK primary care electronic health record (EHR) databases provide population-based, longitudinal data, but their use for rare genetic disease research has not been systematically examined. Through systematic mapping of publications from five UK primary care EHR databases (CPRD, OPCRD, QResearch, SAIL Databank and THIN), we found that only 0.82% (47 of 5754) of studies reported on rare genetic diseases. Of these, 77% (36 of 47) linked to external datasets. Study designs included case-control, cross-sectional and cohort studies. Cohort designs predominated, often with individual-level matched comparators. Case ascertainment was primarily based on routinely recorded diagnostic codes. Most studies examined a single disease, collectively encompassing 23 conditions. There was a skew towards multisystem, neurological, autosomal dominant and single-gene disorders, with relatively higher population frequencies and therapeutic tractability. Rare disease sample sizes ranged from 21 to 5059 (median 392). Important insights were revealed into phenotypic variation, phenotype expansion, complications and management outcomes, including findings not readily identifiable in traditional studies. Examples include higher prevalence of hereditary haemorrhagic telangiectasia in females, consistent with sex-modified phenotypic expression; non-skeletal complications and premature mortality in X-linked hypophosphataemia; and elevated malignancy risk in myotonic dystrophy type 1 with type 2 diabetes, potentially attenuated by metformin. In conclusion, UK primary care EHR databases are markedly underutilised for rare genetic diseases. For many conditions, limited availability of diagnostic codes is a constraint. However, their demonstrated capacity, scale, scope and population representativeness support wider use.

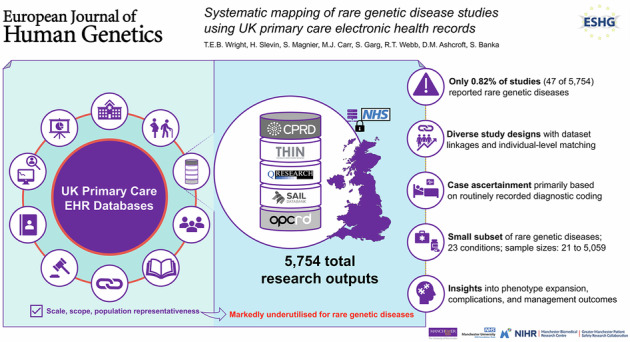

## Introduction

A rare disease is defined in Europe as a disorder that affects fewer than one in 2000 people [1]. The Orphan Drug Act defines a rare disease as a condition affecting fewer than 200,000 individuals living in the United States [2]. There are an estimated 300 million people worldwide living with a rare disease [1], corresponding to 3.5 million individuals in the UK and 30 million in Europe. Most conditions have a genetic aetiology. Rare diseases can manifest with physical and mental health challenges and may impact family dynamics, relationships, education, employment and broader society [3]. Disease-modifying treatments are limited. Studies of rare diseases typically involve small sample sizes, which can lead to statistically underpowered analyses and make conventional study designs unfeasible [4]. Research participant recruitment is often restricted to specialist centres, leading to selected, unrepresentative study populations with limited generalisability [4]. Rare disease registries, although informative, are resource-intensive and difficult to scale and maintain [5].

The UK’s National Health Service (NHS) is one of the largest publicly funded healthcare systems in the world. More than 98% of the UK population is registered with a General Practitioner (GP). A GP is a primary care physician with responsibilities for delivering, coordinating and gatekeeping healthcare. Most NHS consultations occur within primary care. There are three main primary care clinical information systems in the UK: EMIS Web (Optum, formerly EMIS Health), SystmOne (The Phoenix Partnership, TPP) and Vision (Cegedim Healthcare Solutions) [6]. These differ in user interfaces, but are all designed for clinical record keeping and can incorporate correspondence from health and social care providers. Clinical terminologies are based on Read codes, Systematized Nomenclature of Medicine Clinical Terms (SNOMED CT) and vendor-specific codes, reflecting legacy and current coding systems. Prescriptions are aligned with the NHS Dictionary of Medicines and Devices (dm+d).

There are several large, regularly updated national research databases derived from electronic health records (EHR) of patients registered at UK primary care practices. These databases provide access to routinely collected pseudonymised data. Structured data captures patient demographics, registration details, consultations, diagnoses, presentations, investigations, prescriptions and referrals (Supplementary Fig. 1). Examples of UK primary care EHR databases include Clinical Practice Research Datalink (CPRD) [7, 8], Optimum Patient Care Research Database (OPCRD) [9], QResearch [10], The Health Improvement Network (THIN) [11] and Welsh Longitudinal General Practice (WLGP) dataset accessed through Secure Anonymised Information Linkage (SAIL) Databank [12] (Supplementary Table 1). Strengths include their size, population representativeness and breadth of available data [6]. Data linkage can be established at the individual level with other national datasets capturing hospital admissions, outpatient appointments, indices of deprivation measured at the small-area level, and death registrations.

Published evaluations of UK primary care EHR research databases consistently demonstrate comparability with the national population, in terms of age [7, 9, 11], sex [7, 9, 11], ethnicity [13] and socioeconomic position [7, 9]. They have supported research in epidemiology, health economics, risk prediction modelling and randomised controlled trials to generate real-world evidence informing national guidelines [14, 15]. Study eligibility is primarily determined by diagnoses recorded in routinely collected health data, thereby widening participation to individuals who might otherwise be unable or unwilling to take part in traditional research studies [4]. Individual consent is not required; instead, access to pseudonymised data is subject to study-level approval by an independent scientific advisory board and supported by national legal frameworks, including Section 251 of the NHS Act 2006, UK General Data Protection Regulation (GDPR) and the Data Protection Act 2018. Approved research is conducted within a trusted research environment or secure data platform. Stringent governance stipulations are in place to protect privacy and confidentiality, with strict disclosure controls (e.g., not reporting cell counts below five).

UK primary care EHR databases provide a secure and representative platform for conducting health research at scale and appear well-suited to rare genetic diseases (Supplementary Fig. 2). A multistakeholder task force convened by the International Rare Diseases Research Consortium (IRDiRC) highlighted longitudinal primary care records as a potential resource for understanding natural history, supporting diagnosis and improving management [16]. This was also recognised in the UK Department of Health and Social Care Task and Finish Group 2021 report, *The Diagnostic Odyssey in Rare Diseases*, which suggested UK primary care EHR databases could be used to measure temporal changes in diagnostic odyssey [17]. Their broader application, however, remains poorly characterised. This review aimed to systematically identify studies of rare genetic diseases conducted using UK primary care EHR databases, describe their characteristics and synthesise the mapped literature to assess the opportunities and challenges of using these databases for conducting rare genetic disease research.

## Materials and methods

### Data sources, study eligibility criteria, study screening, selection and data collection

The online bibliographies of five major UK primary care EHR research databases (CPRD, OPCRD, QResearch, SAIL Databank and THIN) were accessed on 21 January 2025 via their publicly available URLs. Titles were independently assessed by two clinician investigators using a two-stage procedure. In the first stage, all titles indexed in the bibliographies were screened to identify all potentially relevant studies, including those where eligibility could not be inferred from the title. In the second stage, full-text assessments were undertaken for all articles identified in stage 1 against predefined eligibility criteria. Inclusion criteria were: (i) peer-reviewed publications; (ii) use of one or more of the five specified UK primary care EHR databases (CPRD, OPCRD, QResearch, SAIL Databank and THIN); (iii) study of a germline rare genetic disease catalogued in Orphanet (with a prevalence of <1 in 2000 in Europe); and (iv) findings reported separately for the rare genetic disease. Exclusion criteria were: (i) conditions with non-germline genetic or non-genetic aetiology (e.g., oligogenic, polygenic, multifactorial, somatic, or unknown); and (ii) findings not disaggregated by rare genetic disease (i.e., included only as part of aggregated groups, composite phenotypes, or descriptive counts without disease-specific outcome reporting). No restrictions were imposed on the publication date or study design. Discrepancies were resolved by consensus, with input from the wider study team where required. Summary data were extracted from all eligible publications.

### Data extraction procedure and narrative synthesis

Data from eligible studies were extracted using a standardised form. Study characteristics were recorded, including rare genetic diseases, methodologies, key findings, impact and implications. Each condition was verified and summarised using information from Orphanet (www.orpha.net), a rare disease information portal and ontology. Each Orphanet condition is assigned a unique ORPHAcode mapped to other classification systems, including Online Mendelian Inheritance in Man (OMIM) and the World Health Organization (WHO) International Classification of Diseases, 10th revision (ICD-10). Condition name, ORPHAcode, OMIM codes, ICD-10 codes, inheritance and prevalence estimates were extracted from Orphanet. The WHO online browser tool (https://icd.who.int/browse10/2019/en) was used to document the official ICD-10 terms and index terms. This enabled assessment of whether the rare disease had a specific ICD-10 code or was classified under a broader diagnostic category. ICD-10 remains the diagnostic classification system used in national administrative datasets linked with UK primary care databases, including Hospital Episode Statistics (HES) and Office for National Statistics (ONS) death registrations. Study methodology from all eligible studies was summarised by study design, case ascertainment, case definition, comparator details, sample size, study period, datasets, outcome domains, statistical analyses, key findings, implications and impact. The use of ethnicity and area-level deprivation data was recorded. Patient and public involvement and engagement (PPIE) activities were documented. Funding declarations for each publication were collated. Together, this informed a narrative synthesis of current and potential applications of UK primary care EHR databases for rare genetic disease research.

## Results

### Rare genetic disease studies are underrepresented in research outputs from UK primary care EHR databases

**Fig. 1:**
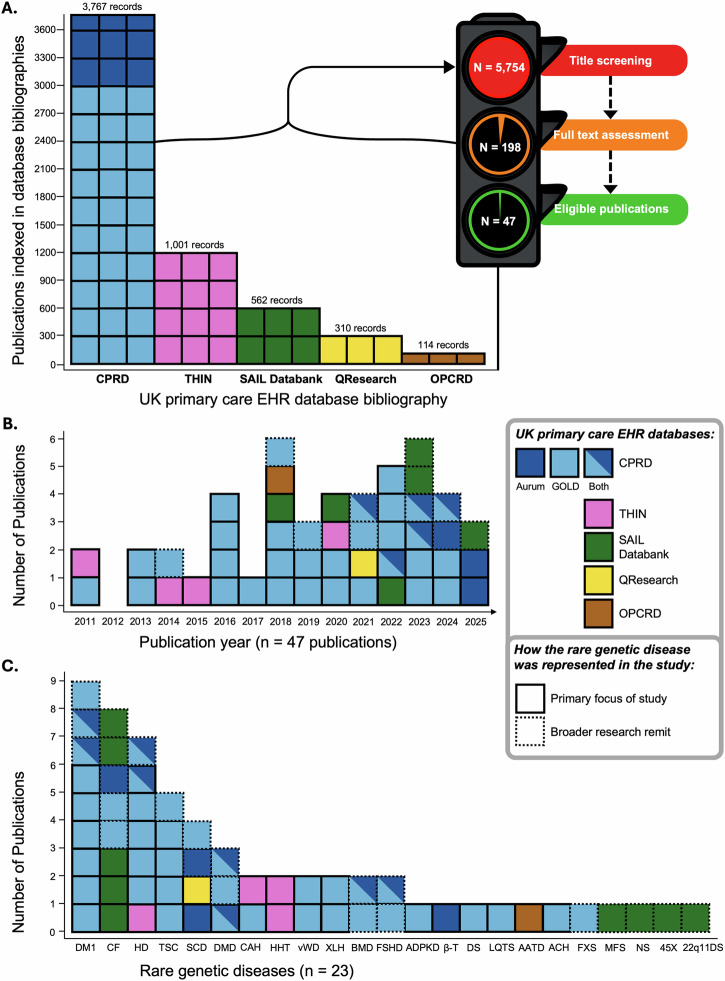
Identification and characteristics of publications reporting rare genetic diseases using UK primary care electronic health record research databases. **A** Bibliographic identification and screening outcomes. Stacked squares represent indexed research outputs from five UK primary care databases as of 21 January 2025 (one square is 300 records), coloured by database. The adjacent traffic light summarises title screening (*n* = 5754), full text assessment (*n* = 198), and eligible peer-reviewed publications (*n* = 47). **B** Temporal distribution of eligible publications (one square is one publication), coloured by database and plotted by publication year. Solid outlines indicate studies with a primary focus on the rare genetic disease; dashed outlines indicate studies in which the condition formed part of a broader research remit. **C** Condition-level mapping of eligible publications. Each square represents a single publication and is repeated for every condition reported; multi-condition studies therefore contribute multiple squares. Conditions are ordered from most to least frequently reported: DM1, myotonic dystrophy type 1; CF cystic fibrosis; HD Huntington’s disease; TSC tuberous sclerosis complex; SCD sickle cell disease; DMD Duchenne muscular dystrophy; CAH congenital adrenal hyperplasia; HHT hereditary haemorrhagic telangiectasia; vWD hereditary von Willebrand disease; XLH X-linked hypophosphataemia; BMD Becker muscular dystrophy; FSHD facioscapulohumeral muscular dystrophy; ADPKD autosomal dominant polycystic kidney disease; β-T beta-thalassaemia; DS Dravet syndrome; LQTS congenital long QT syndrome; AATD alpha-1 antitrypsin deficiency; ACH achondroplasia; FXS Fragile X syndrome; MFS Marfan syndrome; NS Noonan syndrome; 45X, Turner syndrome; 22q11.2DS 22q11.2 deletion syndrome.

First, we set out to identify all peer-reviewed publications reporting rare genetic diseases from five major longstanding UK primary care EHR databases: CPRD, OPCRD, QResearch, SAIL Databank and THIN. In total, these database bibliographies indexed 5754 studies published between 1987 and 2025 (Supplementary Fig. 3). Title screening yielded 198 articles for full-text review. Of these, 152 were excluded. Reasons included failure to report rare genetic diseases separately. One additional publication was identified from the reference list of an eligible paper. In total, 47 articles reported rare genetic diseases, corresponding to 0.82% of all indexed research outputs from the five databases reviewed (Fig. 1A).

The most frequently used database was CPRD (*n* = 35; 0.93% of CPRD publications), followed by SAIL Databank (*n* = 6; 1.07%), THIN (*n* = 4; 0.40%), OPCRD (*n* = 1; 0.88%) and QResearch (*n* = 1; 0.32%) (Table 1). All eligible studies were published from 2011 onwards (Fig. 1B). Most studies used a single UK primary care EHR database (42 of 47). Five studies combined CPRD Gold and CPRD Aurum [18–22], which differ by EHR system and population coverage [7, 8] (Supplementary Table 1). Three studies also used international data [23–25], including analysis of the MarketScan USA claims database in parallel with CPRD [23], a SAIL Databank study forming part of the Establishing a linked European Cohort of Children with Congenital Anomalies (EUROlinkCAT) consortium [24] and a study conducted in Wales and Denmark [25]. Public funders based in the UK, USA, Canada and the EU supported more than half of the studies (25 of 47), either alone (*n* = 19) or in combination with charitable or industry funding (*n* = 6) (Supplementary Table 2). Of the remaining studies, fourteen were funded exclusively by industry, seven exclusively by charitable organisations and one reported no dedicated funding.

**Table 1:** Summary of publications from UK primary care electronic health record databases reporting rare genetic diseases, by primary care database, condition and outcome domains.

Author year [reference]	Database	Rare genetic disease	Outcome domains
Sackley 2011 [33]	THIN	Huntington’s disease^a^	Prevalence, incidence and prescribed medication
Patch 2011 [26]	CPRD Gold	ADPKD^a^	Prescribed antihypertensives, renal replacement and all-cause mortality
Douglas 2013 [27]	CPRD Gold	Juvenile-onset Huntington’s disease^a^	Prevalence, incidence and prescribed medication
Evans 2013 [28]	CPRD Gold	Adult-onset Huntington’s disease^a^	Prevalence
Pouwels 2014 [34]	CPRD Gold	BMD; DMD; DM1; FSHD^b^	Risk of fractures
Donaldson 2014 [36]	THIN^c^	HHT^a^	Prevalence
Donaldson 2015 [37]	THIN^c^	HHT^a^	Complications, comorbidities and all-cause mortality
Wexler 2016 [29]	CPRD Gold	Adult-onset Huntington’s disease^a^	Incidence
Kingswood 2016 [40]	CPRD Gold^c^	TSC^a^	Complications, comorbidities and all-cause mortality
Kingswood 2016 [41]	CPRD Gold^c^	TSC^a^	Healthcare utilisation and costs
Kingswood 2016 [42]	CPRD Gold^c^	TSC with renal manifestations^a^	Healthcare utilisation and costs
Shepherd 2017 [43]	CPRD Gold^c^	TSC with epilepsy^a^	Healthcare utilisation and costs
Wang 2018 [44]	CPRD Gold^c^	Myotonic dystrophy type 1^a^	Risk of skin cancer
Jenkins-Jones 2018 [45]	CPRD Gold^c^	Congenital adrenal hyperplasia^a^	Medication adherence, healthcare costs, depression and all-cause mortality
Soriano 2018 [30]	OPCRD	Alpha-1 antitrypsin deficiency^a^	Genetic testing patterns and characteristics of test positive patients
Schlüter 2018 [25]	SAIL Databank^c^	Cystic fibrosis^a^	Birth weight and gestational age at delivery
Alsaggaf 2018 [46]	CPRD Gold^c^	Myotonic dystrophy type 1^a^	Incidence of cancer
Rassen 2018 [23]	CPRD Gold	Cystic fibrosis^b^	Impact of lookback on prevalence and incidence
Kuan 2019 [47]	CPRD Gold^c^	Cystic fibrosis; sickle cell disease^b^	Prevalence and incidence
Alsaggaf 2019 [63]	CPRD Gold^c^	Myotonic dystrophy type 1^a^	Incidence of benign tumours
Wolfson 2019 [31]	CPRD Gold	Myotonic dystrophy type 1^a^	All-cause mortality
Tresoldi 2020 [38]	THIN^c^	Congenital adrenal hyperplasia^a^	Risk of infections and prescribed antimicrobial medication
Hawley 2020 [64]	CPRD Gold^c^	X-linked hypophosphataemia^a^	Prevalence, all-cause mortality
Alsaggaf 2020 [48]	CPRD Gold^c^	Myotonic dystrophy type 1^a^	Comorbid diabetes mellitus, prescribed metformin and cancer incidence
Griffiths 2020 [57]	SAIL Databank^c^	Cystic fibrosis^a^	Case validation from routinely collected population health data against registry
Clift 2021 [56]	QResearch^c^	Sickle cell disease^a^	COVID-19-related hospitalisation and cause-specific mortality
Carey 2021 [18]	CPRD Gold and Aurum^c^	BMD; DMD; DM1; FSHD^b^	Prevalence and incidence trends
Tyrer 2021 [62]	CPRD Gold^c^	Fragile X syndrome; TSC^b^	All-cause/cause-specific mortality
Hawley 2021 [39]	CPRD Gold^c^	X-linked hypophosphataemia^a^	Comorbidities
Schlüter 2022 [58]	SAIL Databank^c^	Cystic fibrosis^a^	Educational attainment and special educational needs designation
Alothman 2022 [20]	CPRD Gold and Aurum^c^	Huntington’s disease^a^	Cause-specific mortality from suicide
Furby 2022 [61]	CPRD Gold^c^	Adult-onset Huntington’s disease^a^	Prevalence, incidence, comorbidities, prescribed medication, interventions and cause-specific/all-cause mortality
Hagberg 2022 [49]	CPRD Gold^c^	Hereditary von Willebrand disease^a^	Heavy menstrual bleeding and hysterectomy risk
Pickrell 2022 [50]	CPRD Gold^c^	Dravet syndrome^a^	Prevalence, healthcare utilisation, prescribed medication and all-cause mortality
Carey 2023 [19]	CPRD Gold and Aurum^c^	Myotonic dystrophy type 1^b^	Comorbidities and recent infections
Hagberg 2023 [32]	CPRD Gold	Hereditary von Willebrand disease^a^	Prescribed medication for anxiety and depression
Pimenta 2023 [51]	CPRD Gold^c^	Achondroplasia^a^	Complications, healthcare utilisation and cause-specific/all-cause mortality
MacRae 2023 [59]	SAIL Databank^c^	Cystic fibrosis^b^	Multimorbidity and all-cause mortality (differences by dataset)
Divin 2023 [24]	SAIL Databank^c^	Noonan syndrome; Turner syndrome; 22q11.2DS^b^	Prescribed anti-asthmatic medication
Broomfield 2023 [21]	CPRD Gold and Aurum^c^	Duchenne muscular dystrophy^a^	Age at DMD-related clinical milestones: prescribed corticosteroids, spinal surgery, cardiomyopathy, ventilation and all-cause mortality
Dedman 2024 [22]	CPRD Gold and Aurum^c^	Huntington’s disease^b^	Prevalence and incidence (CPRD Gold compared with Aurum)
Evans 2024 [35]	CPRD Gold	Congenital long QT syndrome^a^	Development of a diagnostic prediction model
Wang 2024 [52]	CPRD Aurum^c^	Cystic fibrosis; sickle cell disease^b^	Prevalence (differences by dataset)
Alsaggaf 2024 [53]	CPRD Gold^c^	Myotonic dystrophy type 1^a^	Cause-specific mortality
Udeze 2025 [54]	CPRD Aurum^c^	Sickle cell with recurrent VOC^a^	Transfusions, complications, healthcare utilisation and all-cause mortality
Udeze 2025 [55]	CPRD Aurum^c^	Transfusion-dependent beta-thalassaemia^a^	Comorbidities, complications, healthcare utilisation and all-cause mortality
Chiovoloni 2025 [60]	SAIL Databank^c^	Cystic fibrosis; Marfan syndrome^b^	Prevalence and hospital admissions

ADPKD Autosomal dominant polycystic kidney disease; 22q11.2DS 22q11.2 deletion syndrome; BMD Becker muscular dystrophy; DM1 myotonic dystrophy type 1; DMD Duchenne muscular dystrophy; FSHD facioscapulohumeral muscular dystrophy; HHT Hereditary haemorrhagic telangiectasia; TSC tuberous sclerosis complex; VOC vaso-occlusive crisis.

^a^Study with a primary focus on a rare genetic disease.

^b^Study with a broader research remit, where a rare genetic disease was included in the study, but not as the primary focus.

^c^Study using linked datasets: see Supplementary Table 3 for further details.

Next, we examined the use of linked datasets. Eleven studies relied solely on primary care data [23, 26–35]. A further five studies used primary care records linked only to area-level deprivation data [18, 36–39]. Overall, 77% of studies (36 of 47) linked primary care records to one or more external datasets, including hospital, death registrations and area-level deprivation data (Supplementary Table 3). The most frequent linkage was hospital admissions data, used in 26 studies. These were Hospital Episode Statistics Admitted Patient Care (HES APC), which captures all NHS-funded inpatient and day-case admissions in England and the Patient Episode Database for Wales (PEDW). HES APC was used in 20 CPRD studies [19–22, 40–55] and one QResearch study [56]. PEDW was used in five of six SAIL Databank publications [25, 57–60]. Area-level deprivation measures were used in 19 studies [18, 25, 36–39, 44, 46, 48, 52–56, 58–62]. The Index of Multiple Deprivation (IMD) featured in 15 studies: 11 from CPRD [18, 39, 44, 46, 48, 52–55, 61, 62] and four from SAIL Databank [25, 58–60]. The Townsend Deprivation Index was used in three THIN studies [36–38] and one QResearch study [56]. Other core linkages were ONS death registrations in 15 studies [20–22, 40, 45, 46, 48, 50, 51, 53, 56, 61–64] and hospital outpatient data in 10 studies, including Hospital Episode Statistics Outpatients (HES OP) in England for nine CPRD studies [40–43, 45, 50, 51, 54, 55] and the Outpatient Database for Wales in one SAIL Databank study [60].

We then examined the function of linked datasets within included studies. Approximately one third of publications used linked datasets to define the study population using routinely recorded diagnostic records, usually alongside primary care records (Supplementary Table 3). Linked datasets were also frequently used to derive covariates (e.g., IMD) and a broad range of outcome domains, including comorbidities and complications (Table 1). Mortality outcomes were reported in 17 studies, including 11 that linked with the gold-standard ONS death registration dataset [20, 21, 40, 45, 50, 51, 53, 56, 61, 62, 64] and six that did not [26, 31, 37, 54, 55, 59]. A further four studies used ONS death registrations to ascertain the date of death for censoring (i.e., end of follow-up) or cause of death information to support outcome ascertainment [22, 46, 48, 63]. Other common outcomes included rare disease prevalence and incidence (*n* = 14), prescribed medications (*n* = 11) and healthcare utilisation or costs (*n* = 8). Mental health outcomes were the focus of three studies [20, 32, 45]. One SAIL Databank study [58] investigated educational attainment and special educational needs designation using the National Pupil Database (Table 1).

In summary, rare genetic disease research was markedly underrepresented in outputs from five major UK primary care EHR databases, accounting for fewer than 1% of publications. All eligible studies were published from 2011 onwards, with CPRD as the predominant platform and smaller contributions from SAIL Databank, THIN, OPCRD and QResearch. Linked dataset usage broadened the scope of research and outcome domains spanned health [51], education [58] and mortality [53].

### Rare genetic disease studies from UK primary care EHR databases are concentrated on a small number of disorders

Next, we assessed how rare genetic diseases were represented across the literature. Thirty-six of 47 studies had a primary focus on a rare genetic disease (Fig. 1B). The remaining 11 studies had a broader research remit that included a rare genetic disease, but not as the primary focus. For example, a life course study examined age-specific incidence and period prevalence of 308 phenotypes, including cystic fibrosis and sickle cell disease [47]. All 36 articles with a primary focus investigated a single condition. The largest number of genetic rare diseases reported in a single publication was four [18, 34], forming minor components of studies with a broader research remit. In total, 23 rare genetic diseases were studied (Table 2). Twelve conditions featured in multiple publications, and 11 conditions were reported in a single publication (Fig. 1C). Myotonic dystrophy type 1 was the most studied condition with nine associated publications, followed by cystic fibrosis (*n* = 8) and Huntington’s disease (*n* = 7). Together, these three conditions accounted for more than 50% of studies.

**Table 2:** Rare genetic diseases investigated using UK primary care electronic health record research databases, with ORPHAcode, Orphanet prevalence estimates, inheritance patterns, age of onset, and associated publications from this review.

Rare genetic disease	ORPHAcode	Prevalence in Orphanet	Inheritance	Age of onset	Number of publications [references]
Achondroplasia	ORPHA:15	1 in 25,000 LB	AD	Prenatal	One [51]
Alpha-1 antitrypsin deficiency	ORPHA:60	1-5 in 10,000	AR	Adult	One [30]
Autosomal dominant polycystic kidney disease	ORPHA:730	1-5 in 10,000	AD	Adult	One [26]
Becker muscular dystrophy	ORPHA:98895	1-9 in 100,000	XL	Child	Two [18, 34]
Beta-thalassaemia	ORPHA:848	1-9 in 100,000	AR (+ AD)	Child	One [55]
Congenital adrenal hyperplasia	ORPHA:418	1-9 in 100,000	AR	Neonatal	Two [38, 45]
Congenital long QT syndrome^a^	ORPHA:768	1 in 2500 LB	AD (+ AR)	Child/Adult	One [35]
Cystic fibrosis	ORPHA:586	1-5 in 10,000	AR	Infancy	Eight [23, 25, 47, 52, 57–60]
Dravet syndrome	ORPHA:33069	1 in 30,000 LB	AD	Infancy	One [50]
Duchenne muscular dystrophy	ORPHA:98896	1-9 in 100,000	XL	Child	Three [18, 21, 34]
Facioscapulohumeral muscular dystrophy	ORPHA:269	1-9 in 100,000	AD	Adult	Two [18, 34]
Fragile X syndrome	ORPHA:908	1-5 in 10,000	XL	Child	One [62]
Hereditary haemorrhagic telangiectasia	ORPHA:774	1-5 in 10,000	AD	Adult	Two [36, 37]
Hereditary von Willebrand disease	ORPHA:903	1-5 in 10,000	AD (+ AR)	Child	Two [32, 49]
Huntington’s disease	ORPHA:399	1-9 in 100,000	AD	Adult	Seven [20, 22, 27–29, 33, 61]
Marfan syndrome	ORPHA:558	1-5 in 10,000	AD	Child/Adult	One [60]
Myotonic dystrophy type 1	ORPHA:273	1-5 in 10,000	AD	Adult^b^	Nine [18, 19, 31, 34, 44, 46, 48, 53, 63]
Noonan syndrome	ORPHA:648	1-5 in 10,000	AD (+ AR)	Child	One [24]
Sickle cell disease^a^	ORPHA:275752	1-5 in 10,000	AR	Child	Four [47, 52, 54, 56]
Tuberous sclerosis complex	ORPHA:805	1-9 in 100,000	AD	Child	Five [40–43, 62]
Turner syndrome	ORPHA:881	1-5 in 10,000	N/A	Child	One [24]
X-linked hypophosphataemia	ORPHA:89936	1-9 in 100,000	XL	Child	Two [39, 64]
22q11.2 deletion syndrome	ORPHA:567	1 in 4500–10,000 LB	AD	Child	One [24]

AD autosomal dominant; AR autosomal recessive; N/A not applicable; XL X-linked; brackets indicate minor contributory inheritance. Prevalence was recorded as ‘unknown’ in the structured prevalence field of Orphanet for achondroplasia, congenital long QT syndrome, Dravet syndrome and 22q11.2 deletion syndrome; accompanying summary text provided estimates at birth (LB, live birth) which are shown.

^a^Orphanet group-level disorder classification. All other conditions are classified by Orphanet at the disorder-level.

^b^Age of onset is intended to reflect classical presentations. Myotonic dystrophy type 1 is designated as adult onset; however, congenital and childhood-onset forms also occur and are classified as disorder subtypes in Orphanet.

We then mapped conditions to established rare disease identifiers. Aligned with our eligibility criteria, all 23 conditions had an ORPHAcode (Table 2). Three conditions were classified at the group-level (congenital adrenal hyperplasia, congenital long QT syndrome and sickle cell disease) and 20 at the disorder-level. Group-level classification refers to a collection of clinical entities sharing common features, whereas disorder-level classification denotes individual clinical entities for which a definitive clinical diagnosis can be made. The 23 conditions mapped to 82 OMIM codes, reflecting substantial genetic and phenotypic heterogeneity (Supplementary Table 4).

To indicate where each condition lay on the rare spectrum, we extracted Orphanet prevalence estimates. All conditions in the review fell within the two most frequent prevalence bands (1-5 in 10,000 to 1-9 in 100,000). Four conditions had ‘unknown’ in the structured Orphanet prevalence field, but the accompanying summary text provided estimates at birth (Table 2). The lowest prevalence reported in the review was for juvenile Huntington’s disease (onset before 21 years), with a minimum prevalence of 6.77 per million patient-years and period prevalence in 2010 of 1 in 385,000 [27]. Cystic fibrosis was the most prevalent condition, estimated in one study at 5.76 in 10,000 live births, based on ascertainment from multiple datasets [25]. This estimate marginally exceeded the rare disease prevalence threshold; however, as cystic fibrosis remains classified by Orphanet as a rare genetic disease (ORPHA:586), for completeness, we opted to include it in the review. Rare disease sample sizes ranged from 21 to 5059 cases, with a median of 392 (Supplementary Table 3). A recent CPRD study of beta-thalassaemia had a starting population of 11,359, with analysis restricted to 237 individuals with transfusion-dependent disease [55]. To indicate overall scale, we summed the largest sample size reported for each condition, which resulted in a minimum combined sample size of 21,340 (Supplementary Table 5).

To explore factors that may have influenced which conditions were studied, we reviewed the genetic basis, inheritance and age of onset (Supplementary Table 4). Twenty-one of the 23 conditions were single-gene disorders, and two were chromosomal. Thirteen conditions were autosomal dominant, five were autosomal recessive and four were X-linked. Most were early onset conditions, spanning prenatal (achondroplasia), neonatal (congenital adrenal hyperplasia), infancy (cystic fibrosis) and childhood (Duchenne muscular dystrophy). Five were adult-onset conditions, including alpha-1 antitrypsin deficiency, autosomal dominant polycystic kidney disease, facioscapulohumeral muscular dystrophy, hereditary haemorrhagic telangiectasia and myotonic dystrophy type 1. Congenital and childhood-onset forms of myotonic dystrophy type 1 also occur [46], reflecting variable expressivity and trinucleotide repeat expansion size.

Drawing on OMIM, Orphanet, and our own clinical experience, we characterised conditions further by primary phenotypes and affected systems (Supplementary Table 4). Most conditions were multisystem, with diverse primary phenotypes; the most common were developmental (*n* = 6), neuromuscular (*n* = 4) and haematological (*n* = 3). We then assigned the clinical specialty typically responsible for leading care for each condition, recognising variation in service organisation and that care often involves multidisciplinary teams, specialist services and clinical genetics input. Neurology was the lead specialty for seven conditions, followed by haematology for three (Supplementary Table 4). Other clinical specialties included paediatrics, cardiology, endocrinology, respiratory medicine and nephrology.

Finally, noting that targeted therapies may have contributed to research activity, we reviewed established treatments. Of the 23 conditions, 11 had therapies beyond supportive or symptomatic management (Supplementary Table 4). These included replacement-based treatments such as corticosteroid and mineralocorticoid replacement for congenital adrenal hyperplasia, growth hormone therapy for Turner syndrome, von Willebrand factor concentrates for hereditary von Willebrand disease, and plasma-derived augmentation therapy for alpha-1 antitrypsin deficiency, as well as targeted or disease-modifying therapies, such as burosumab for X-linked hypophosphataemia, CFTR modulators for cystic fibrosis, mTOR inhibitors for tuberous sclerosis complex, tolvaptan for autosomal dominant polycystic kidney disease and vosoritide for achondroplasia. Casgevy (exagamglogene autotemcel) has recently become clinically available for transfusion-dependent beta-thalassaemia and sickle cell disease, coinciding with two recent CPRD publications [54, 55]. Exon-skipping therapies for Duchenne muscular dystrophy are approved in the USA and Japan, and preliminary findings from the Huntington’s disease AMT-130 gene therapy trial at 36 months showed promising results.

In summary, outputs from UK primary care EHR databases span a broad range of rare genetic diseases, but research activity is skewed towards multisystem, neurological, autosomal dominant, single-gene disorders with relatively higher population frequencies and established or emerging treatments.

### Cohort designs predominate in rare disease studies using UK primary care records

To demonstrate the range of methodological approaches used, we summarised study designs (Supplementary Table 3). Most studies used cohort designs (*n* = 38; 81%). The remainder were case-control studies (*n* = 3), cross-sectional studies (*n* = 2) and methodological evaluations (*n* = 4). Among the 38 cohort studies, 26 included a comparator group: 19 used individual-level matched comparators, six used non-matched internal comparators and one used an external dataset for comparison. Next, we examined individual-level matching strategies, implemented in cohort, case-control and cross-sectional designs. Age and sex matching were used in all matched studies (*n* = 23). Nineteen studies also matched on primary care practice. Less common matching variables were geographical region, ethnicity and deprivation. A case-control study used propensity score matching based on age, sex, BMI, smoking status, ethnicity and primary care practice [35]. Where reported, index dates for matching were typically aligned to the earliest diagnostic record, calendar year, or GP registration. Some studies used data completeness eligibility thresholds, such as a minimum primary care registration period [23] or evidence of healthcare activity [41, 42]. The number of comparators matched to each rare disease case ranged from 1:2 to 1:40, with 1:5 being the most common in six studies, followed by 1:20 in five studies. The highest ratio of 1:40 was from a case-control study using risk-set sampling [20].

We then appraised routes of ascertainment (i.e., data sources used to define the rare disease study population). In 46 of 47 studies, eligibility was defined by primary care diagnostic codes, with 28 exclusively using primary care data and 18 studies also permitting diagnostic records from linked datasets (Supplementary Table 3). One study exclusively used the Congenital Anomaly Register and Information Service (CARIS) in Wales [24]. Where secondary care administrative records contributed to case ascertainment, this was based on ICD-10-coded hospital admissions from HES APC in England and PEDW in Wales. We evaluated whether the 23 conditions from the review mapped to specific ICD-10 descriptors explicitly named for each disorder. Around half of the conditions (11 of 23) had a dedicated ICD-10 code and the remaining 12 were classified into broader diagnostic groups (Supplementary Table 4).

We then examined how rare genetic diseases were defined. Most studies used simple case definitions based on the presence of at least one routinely recorded diagnostic code (Supplementary Table 3). More complex case definitions incorporated additional criteria such as prescriptions (e.g., corticosteroids for congenital adrenal hyperplasia [45]), demographic restrictions (e.g., restricted Duchenne muscular dystrophy cohort to males aged under 50 years [21]) and exclusion rules (e.g., hereditary von Willebrand disease presumed in the absence of diagnostic records for conditions associated with acquired disease [32, 49]). Other studies used multiple criteria or algorithm-based definitions [39, 64]. Event-based criteria were used to define recurrent vaso-occlusive crises in sickle cell disease [54] and transfusion-dependent beta-thalassaemia [55]. Some studies used sensitivity analyses or validation exercises to assess the robustness of case definitions. One study assessed the impact of applying a broader case definition for hereditary haemorrhagic telangiectasia [36]. Another tested alternative definition for Duchenne muscular dystrophy requires at least two diagnostic records or an ICD-10 code in HES APC [21]. A further study validated Huntington’s disease diagnoses by reviewing free-text entries to exclude misclassification of unaffected individuals with a family history [61]. This approach is no longer feasible under current UK GDPR restrictions [6, 7]. Only one study undertook external validation, against the UK Cystic Fibrosis Registry and found that combining diagnostic records from primary care and linked datasets improved sensitivity with minimal loss of specificity [57].

Where diagnostic codes were unavailable, broad, or infrequently used, adapted case definition strategies were used. For example, X-linked hypophosphataemia was defined by combining broad skeletal phenotypic descriptors, biochemical results and prescriptions [39, 64]. Likelihood grading was independently undertaken by two national clinical experts in familial hypophosphataemia, with high inter-grader agreement. Similarly, probable Dravet syndrome was defined by a diagnostic record of epilepsy together with a prescription of stiripentol or potassium bromide [50]. A third example distinguished achondroplasia from hypochondroplasia, which shares ICD-10 code Q77.4, when the age of diagnosis was before two, or height was within the achondroplasia reference range [51].

Two studies suggested probable misclassification of carriers of autosomal recessive and X-linked conditions. Cystic fibrosis was found to have a bimodal distribution for diagnostic age, with peaks in early childhood and at 30 years [47]. The second peak was attributed to carriers, likely misclassified following parental genetic testing or during family planning. Another study found that 324 females had a diagnostic record of Duchenne muscular dystrophy, which far exceeds those expected to have a classical phenotype [21]. This study also reported 12 males over the age of 50 with a record of Duchenne muscular dystrophy. These findings were considered clinically implausible, but only comprised 1.1% of the cohort [21]. Sensitivity analyses showed no material effect on the findings.

Overall, most studies used cohort designs and primary care diagnostic codes to delineate rare genetic disease study populations. Case definitions were adapted when coding was limited.

### UK primary care EHR databases provide insights into the epidemiology, natural history and clinical management of rare genetic diseases

To illustrate the capabilities of UK primary care EHR databases, we synthesised key findings, implications and impact of studies (Supplementary Table 3). We provide three exemplars to demonstrate the breadth of insights achievable.

The first exemplar showcases the capacity to investigate population-level diagnostic patterns and phenotypic variation. Using THIN, one study estimated hereditary haemorrhagic telangiectasia prevalence annually by age, sex, geographical region and socioeconomic position [36]. The study reported higher UK prevalence than previously recognised and identified diagnostic disparities. There was marked female predominance despite the condition’s autosomal dominant inheritance [36]. These findings suggest sex-modified phenotypic expression and are consistent with international liver transplant registry reports, in which most hereditary haemorrhagic telangiectasia-related liver transplant recipients are female [65]. Registry studies also report that females have more severe hepatic and pulmonary arteriovenous malformations and undergo more invasive procedures [65].

The second exemplar illustrates the potential for multisystem phenotyping in ultra-rare diseases. A CPRD study reported premature mortality in X-linked hypophosphataemia compared with matched comparators [64]. A follow-up study examined 273 resource-intensive comorbidities across 15 disease categories and found a higher prevalence of endocrine and neurological disorders [39]. Four individual comorbidities occurred at least twice as often, including depression, which remained significant after multiple testing correction. These findings extend the recognised phenotype of X-linked hypophosphataemia beyond its classical skeletal manifestations.

The third exemplar illustrates how nationally representative rare disease cohorts can quantify risk and support evaluation of risk-modifying treatments. Using CPRD, a matched cohort study of 1061 individuals with myotonic dystrophy type 1 reported a five-fold increased risk of basal cell carcinoma versus 15,119 matched comparators [44]. Non-melanoma skin cancer is not typically recorded in cancer registries; therefore, this analysis would be challenging to replicate using alternative datasets. Additional studies investigating myotonic dystrophy type 1 identified increased risks of benign [63] and malignant tumours [46], with evidence that age at diagnosis of myotonic dystrophy type 1 appears to modify cancer susceptibility. A further study suggested that metformin may attenuate cancer risk in individuals with myotonic dystrophy type 1 who also had type 2 diabetes mellitus [48].

Finally, we examined the use of equity-related variables and PPIE in studies of rare genetic diseases using UK primary care EHR databases. Area-level deprivation appeared in 19 studies [18, 25, 36–39, 44, 46, 48, 52–56, 58–62]. By contrast, ethnicity data were only reported in seven studies [35, 47, 52, 54–56, 60]. No studies reported PPIE activities in their publications.

## Discussion

To our knowledge, this is the first detailed examination of how UK primary care EHR databases have been used to study rare genetic diseases. Nonetheless, some limitations should be acknowledged. Eligibility was restricted to peer-reviewed publications explicitly reporting the use of five named UK primary care EHR databases. Consequently, research using other data sources, including newer national primary care EHR data initiatives (e.g., OpenSAFELY), regional datasets and non-UK data resources, fell outside the scope of this review. Study identification relied on database bibliographies and indexing practices, which may have resulted in some relevant studies being missed. Restricting included outputs to peer-reviewed articles published in academic journals may have overlooked reports produced by pharmaceutical companies around drug development and regulatory approval. Finally, because the review was designed specifically to map published studies of rare genetic diseases, the findings may not be generalisable to rare diseases with non-genetic aetiologies.

Notwithstanding these limitations, our review identified several important findings. We show that despite their demonstrated capacity, versatility, scale and population representativeness, UK primary care EHR databases are markedly underutilised for rare genetic diseases (Fig. 1). The low volume of research is particularly striking when contrasted with the broader research activity of these databases [15]. A scientometric analysis of CPRD, THIN and QResearch from 1995 to 2015 found that each of the top 30 conditions accounted for at least 3% of total research outputs [15]. In contrast, only seven rare genetic disease studies were published over the same 20-year period [26–28, 33, 34, 36, 37], representing less than 0.4% of outputs and a 32-fold disparity relative to diabetes mellitus publications [15]. This imbalance is striking given that the annual economic costs to society  of 373 rare diseases estimated in a USA-based study was US$2.2 trillion, compared with US$3.4 trillion for common diseases such as diabetes mellitus, cardiovascular disease and cancer [3].

Cystic fibrosis, myotonic dystrophy type 1 and Huntington’s disease together accounted for more than half of the studies in our review. Their prominence likely reflects a combination of factors, including diagnostic visibility, clinical familiarity, longstanding recognition in medical practice and availability of codes in routinely collected health data. Reuse of existing codelists and repeated outputs from the same research groups [44, 46, 48, 63] also contributed to condition recurrence in the literature. Our findings align with the *NIHR Rare Diseases Research Landscape Report* [66], which described a skewed distribution of rare disease research activity during 2016 to 2021, in which a small number of rare conditions accounted for a large share of research and most had no visible research [66]. UK primary care databases were largely overlooked in this report. One illustrative example was Mendelian’s MendelScan, an artificial intelligence case-finding platform for rare diseases using data from approximately 50 NHS primary care practices in England [66]. At the time of the report, this valuable initiative was substantially smaller in scale than the databases described in our review (Supplementary Table 1) but illustrates an additional application not otherwise captured in our review.

Findings from the IRDiRC *State of Play Report 2019-2021* were also consistent [67], with 35% of conditions in our review (8 of 23) also appearing among the top 20 most researched non-neoplastic rare disorders worldwide. Rare neurological disorders accounted for the largest share of research globally (37%), mirroring the distribution observed in our review (Table 2). In the NIHR report, cystic fibrosis was given as an exemplar condition with high research activity attributed to its relatively high prevalence and biologically tractable therapeutic targets [66]. Consistent with this, around half of the conditions in our review had established treatments (Supplementary Table 4). Among the 36 studies with a primary focus on a rare genetic disease, pharmaceutical companies were the sole funders of 13 studies spanning nine conditions (Supplementary Table 2). This supports the hypothesis that therapeutic tractability is a key factor shaping current research activity. Extending the use of UK primary care EHR databases to more rare diseases is likely to require prioritisation and coordinated investment.

A major strength of UK primary care EHR databases is that they allow rare disease cohorts to be defined within a representative population-based setting and followed up longitudinally. Delineating comparator cohorts drawn from the same source population is another key strength. This was reflected in our review by the predominance of matched cohort designs (Supplementary Table 3). For rarer outcomes, alternative study designs may be more suitable; for example, death by suicide in Huntington’s disease was examined using a case-control design with risk-set sampling [20], as a matched cohort design would have been underpowered. Databases are most informative when conditions and outcome phenotypes can be identified with confidence in primary care and linked datasets. All conditions in our review had Orphanet prevalence estimates ranging from 1 in 2000 to 1 in 100,000 (Supplementary Table 5), suggesting conditions in this prevalence range may be feasible to study. Coding specificity varies considerably between conditions and case definitions may be strengthened using corroborative evidence for condition-specific features [51, 64], diagnostic confirmation in linked hospital data [21] and, where possible, external validation [57]. Sensitivity analyses can assess the robustness of findings to alternative case definitions [21, 33, 36]. Databases are currently less informative for questions requiring deep phenotyping or outcomes poorly captured in routinely collected health data. By contrast, they support research questions pertaining to rare disease prevalence and incidence, comorbidities, prescribed medications, health economics and mortality (Table 1). Their effective use requires expertise in epidemiology, statistical analyses, coding frameworks, clinical interpretation and familiarity with NHS healthcare delivery. Approval processes, access requirements and timescales vary across databases [6] and may present practical barriers to their wider use in rare disease research.

All studies in the review with a primary focus on a rare genetic disease examined a single condition (Supplementary Table 3). In contrast, other studies concurrently investigated up to 308 conditions [47], demonstrating technical feasibility to investigate multiple rare diseases. The first attempt to enumerate all rare diseases identifiable in the UK population health datasets was published in February 2025 [68]. This study used the General Practice Extraction Service Extract for Pandemic Planning and Research (GDPPR, data from 98% of NHS primary care practices in England) and NHS hospital data to estimate prevalence and COVID-19-related mortality risk for all identifiable rare diseases [68]. Rare diseases were defined as Orphanet entities that mapped to ICD-10 or SNOMED CT codes with high specificity. Using this approach, 331 rare diseases were identified [68]. Eight non-mutually exclusive categories derived from Orphanet’s classification tags informed subgroup analysis. The genetic disease group was heterogeneous, encompassing high-penetrance Mendelian genetic disorders (e.g., Smith-Magenis syndrome), clinical syndromes with variable genetic contribution (e.g., Lennox-Gastaut syndrome) and several predominantly non-genetic entities and descriptive diagnoses (e.g., interatrial communication (i.e., atrial septal defect), congenital laryngomalacia and isolated plagiocephaly) [68]. The infrastructure to scale rare genetic disease research using routinely collected UK population health data exists, but case definitions, disease groupings and interpretation require careful clinical consideration.

Only seven of the 23 conditions from our review were identified among the 331 rare diseases in the GDPPR-linked study [68]. Achondroplasia was designated as having a highly specific ICD-10 code (Q77.4); however, this code is shared with hypochondroplasia [51]. This limitation was addressed in a CPRD study by refining the eligibility criteria using age at diagnosis and height data [51]. Other studies in our review adapted case definitions where diagnostic codes were unavailable, inconsistently applied, or ambiguous [39, 51, 64]. The flexibility to refine case definitions using clinical logic is a major strength of UK primary care research. Case definitions require bespoke curation using established codelist development methodologies, including systematic searching of data dictionaries with clinician oversight. Scaling this approach to multiple rare diseases is challenging. Using Orphanet mappings to clinical terminologies is pragmatic and as Orphadata is updated annually, these will expand over time. However, application and interpretation should be informed by clinicians working in genomic medicine.

Improving rare genetic disease research using primary care data depends largely on the suitability of clinical vocabularies. SNOMED CT is the core terminology used in UK general practice, but representation of rare diseases is limited [68]. NHS hospital administrative datasets use ICD-10 codes, which also lack precision for most rare diseases. The planned transition to ICD-11 introduces closer alignment with Orphanet. Embedding ORPHAcodes in existing clinical information systems and national administrative datasets could improve rare disease specificity and interoperability. Many rare genetic diseases are diagnosed in hospital outpatient settings; however, diagnoses are recorded for only a minority of outpatient appointments in routine datasets. Mandating this would strengthen case ascertainment. A critical next step is the integration of genomic data. None of the studies in our review linked primary care records to genomic data, reflecting this absence in standard linkage schemes available in UK primary care databases. Feasibility has nevertheless been shown in SAIL Databank, where primary care records were linked to whole exome sequencing data to investigate epilepsy outcomes [69]. Establishing a secure linkage between primary care records and data from NHS genomic medicine services would enable cohort validation and genotype-phenotype studies.

Historically, UK primary care databases relied on networks of contributing practices. SAIL Databank was the only database in our review designed to capture a national population, with primary care records available for around 90% of Wales (Supplementary Table 1). The COVID-19 pandemic prompted the development of whole population analytic platforms in England, including GDPPR, CVD-COVID-UK/COVID-IMPACT and OpenSAFELY. Although initially focused on COVID-19, their remit is expanding, creating opportunities for rare disease research [68]. In April 2025, the UK Government announced a £600 million partnership to establish the Health Data Research Service as a single secure access point to linked NHS data for approved research. On a European level, primary care data from CPRD contributes to the Data Analysis and Real World Interrogation Network (DARWIN EU®), coordinated by the European Medicines Agency. THIN also includes UK primary care data alongside health data from other European countries [6]. The European Health Data Space (EHDS) is a newly adopted regulation intended to establish a common framework for health data reuse in research and policy.

Federated approaches, as illustrated by the EUROlinkCAT study [24], enable secure local analyses with aggregation of results across partners. In Wales, the EUROlinkCAT cohort was derived from CARIS and linked primary care prescribing data through SAIL Databank [24]. In England, comparable registry infrastructure is provided by the National Disease Registration Service (NDRS), which manages the National Congenital Anomaly and Rare Disease Registration Service (NCARDRS). Rare disease representation within NCARDRS is limited and primary care linkage is not currently available. NDRS strategic priorities include expanding rare disease registration and developing algorithms to identify rare diseases in routinely collected datasets. Consistent with this, the Registration of Complex Rare Diseases—Exemplars in Rheumatology (RECORDER) project validated HES ICD-10 coded rare autoimmune diseases against clinical records [70].

Progress in this field must align with principles of equity and patient and public partnership. The absence of ethnicity reporting in most studies likely reflects historically incomplete recording in primary care. Ethnicity recording has improved and consolidating information from multiple linked data sources increases completeness [13]. Socioeconomic measures available within UK primary care databases are informative, but area-level measurements have recognised limitations. Primary care EHR databases can support the identification of disparities in rare disease diagnoses, treatment and outcomes, contributing to the UK Rare Diseases Framework’s vision to improve the quality and availability of care and address health inequalities. No studies in the review reported patient or public involvement, highlighting an important gap in current research practice. Embedding PPIE throughout study design, implementation and dissemination will ensure research priorities reflect the experiences of individuals and families living with rare diseases.

In conclusion, UK primary care EHR databases provide routinely collected, population-based, longitudinal data that are linkable to national healthcare and mortality datasets. Their scale, scope and population representativeness support natural history studies for rare genetic diseases. Despite this, they are markedly underutilised. For many conditions, the limited availability of diagnostic codes in routinely collected health data is a major constraint. Strengthening clinical coding vocabularies, expanding data initiatives to achieve whole population coverage, establishing standard data linkage with NHS genomic medicine services, enabling federated analysis and embedding patient partnerships will be key to unlocking their full potential.

## Supplementary information

Supplementary Figures 1-3

Supplementary Tables 1-5

## Data Availability

All data supporting the findings of this review are included in this article and its supplementary files. No new primary datasets were generated.

## References

[1] Nguengang Wakap S, Lambert DM, Olry A, Rodwell C, Gueydan C, Lanneau V, et al. Estimating cumulative point prevalence of rare diseases: analysis of the Orphanet database. Eur J Hum Genet. 2020;28:165–73. 10.1038/s41431-019-0508-0. 31527858 10.1038/s41431-019-0508-0 PMC6974615

[2] Herder M. What is the purpose of the Orphan Drug Act? PLoS Med. 2017;14:e1002191. 10.1371/journal.pmed.1002191. 28045908 10.1371/journal.pmed.1002191 PMC5207521

[3] The Lancet. Hope for rare diseases. The Lancet. 2024;404:1701. 10.1016/S0140-6736(24)02414-0 10.1016/S0140-6736(24)02414-0 39488398

[4] Griggs RC, Batshaw M, Dunkle M, Gopal-Srivastava R, Kaye E, Krischer J, et al. Clinical research for rare disease: opportunities, challenges, and solutions. Mol Genet Metab. 2009;96:20–6. 10.1016/j.ymgme.2008.10.003. 19013090 10.1016/j.ymgme.2008.10.003 PMC3134795

[5] Hageman IC, Van Rooij IALM, De Blaauw I, Trajanovska M, King SK. A systematic overview of rare disease patient registries: challenges in design, quality management, and maintenance. Orphanet J Rare Dis. 2023;18:106. 10.1186/s13023-023-02719-0. 37147718 10.1186/s13023-023-02719-0 PMC10163740

[6] Edwards L, Pickett J, Ashcroft DM, Dambha-Miller H, Majeed A, Mallen C, et al. UK research data resources based on primary care electronic health records: review and summary for potential users. BJGP Open. 2023;7:BJGPO.2023.0057. 10.3399/BJGPO.2023.0057. 37429634 10.3399/BJGPO.2023.0057 PMC10646196

[7] Wolf A, Dedman D, Campbell J, Booth H, Lunn D, Chapman J, et al. Data resource profile: Clinical Practice Research Datalink (CPRD) Aurum. Int J Epidemiol. 2019. 10.1093/ije/dyz034 10.1093/ije/dyz034 PMC6929522 30859197

[8] Sanchez-Santos MT, Axson EL, Dedman D, Delmestri A. Data resource profile update: CPRD GOLD. Int J Epidemiol. 2025;54:dyaf077. 10.1093/ije/dyaf077. 40499193 10.1093/ije/dyaf077 PMC12158158

[9] Lynam A, Curtis C, Stanley B, Heatley H, Worthington C, Roberts EJ, et al. Data-Resource Profile: United Kingdom Optimum Patient Care Research Database. Pragmatic Obs Res. 2023;14:39–49. 10.2147/POR.S395632. 10.2147/POR.S395632 PMC10150735 37138785

[10] Hippisley-Cox J, Stables D, Pringle M. QRESEARCH: a new general practice database for research. J Innov Health Inf. 2004;12:49–50. 10.14236/jhi.v12i1.108. 10.14236/jhi.v12i1.108 15140353

[11] Blak B, Thompson M, Dattani H, Bourke A. Generalisability of The Health Improvement Network (THIN) database: demographics, chronic disease prevalence and mortality rates. J Innov Health Inf. 2011;19:251–5. 10.14236/jhi.v19i4.820. 10.14236/jhi.v19i4.820 22828580

[12] Jones KH, Ford DV, Thompson S, Lyons RA. A Profile of the SAIL Databank on the UK Secure Research Platform. Int J Popul Data Sci. 2019;4. 10.23889/ijpds.v4i2.1134 10.23889/ijpds.v4i2.1134 PMC8142954 34095541

[13] Shiekh S, Harley M, Ghosh R, Ashworth M, Myles P, Booth H, et al. Completeness, agreement, and representativeness of ethnicity recording in the United Kingdom’s Clinical Practice Research Datalink (CPRD) and linked Hospital Episode Statistics (HES). Popul Health Metr. 2023;21:3. 10.1186/s12963-023-00302-0. 36918866 10.1186/s12963-023-00302-0 PMC10013294

[14] Leahy TP, Ramagopalan S, Sammon C. The use of UK primary care databases in health technology assessments carried out by the National Institute for Health and Care Excellence (NICE). BMC Health Serv Res. 2020;20:675. 10.1186/s12913-020-05529-3. 32698805 10.1186/s12913-020-05529-3 PMC7374907

[15] Vezyridis P, Timmons S. Evolution of primary care databases in the UK: a scientometric analysis of research output. BMJ Open. 2016: e012785. 10.1136/bmjopen-2016-012785 10.1136/bmjopen-2016-012785 PMC5073525 27729352

[16] Baynam G, Hartman AL, Letinturier MCV, Bolz-Johnson M, Carrion P, Grady AC, et al. Global health for rare diseases through primary care. Lancet Glob Health. 2024;12:e1192–9. 10.1016/S2214-109X(24)00134-7. 38876765 10.1016/S2214-109X(24)00134-7 PMC13271179

[17] Hay, E, Elmslie, F, Lanyon, P, Cole, T. The Diagnostic Odyssey in rare diseases: a Task and Finish Group report for the Department of Health and Social Care. National Institute for Health Research; 2022. 10.3310/nihropenres.1115171.1.

[18] Carey IM, Banchoff E, Nirmalananthan N, Harris T, DeWilde S, Chaudhry UAR, et al. Prevalence and incidence of neuromuscular conditions in the UK between 2000 and 2019: a retrospective study using primary care data. PLoS ONE. 2021;16:e0261983. 10.1371/journal.pone.0261983 10.1371/journal.pone.0261983 PMC8719665 34972157

[19] Carey I, Nirmalananthan N, Harris T, DeWilde S, Chaudhry U, Limb E, et al. Prevalence of co-morbidity and history of recent infection in patients with neuromuscular disease: a cross-sectional analysis of United Kingdom primary care data. PLoS One. 2023;18:e0282513. 10.1371/journal.pone.0282513. 36857388 10.1371/journal.pone.0282513 PMC9977045

[20] Alothman D, Marshall CR, Tyrrell E, Lewis S, Card T, Fogarty A. Risk of mortality from suicide in patients with Huntington’s disease is increased compared to the general population in England. J Neurol. 2022;269:4436–9. 10.1007/s00415-022-11085-z. 35344078 10.1007/s00415-022-11085-z PMC9293836

[21] Broomfield J, Abrams K, Latimer N, Guglieri M, Rutherford M, Crowther M. Natural history of Duchenne muscular dystrophy in the United Kingdom: a descriptive study using the Clinical Practice Research Datalink. Brain Behav. 2023;13:e3331. 10.1002/brb3.3331. 37957895 10.1002/brb3.3331 PMC10726817

[22] Dedman D, Williams R, Bhaskaran K, Douglas IJ. Pooling of primary care electronic health record (EHR) data on Huntington’s disease (HD) and cancer: establishing comparability of two large UK databases. BMJ Open. 2024;14:e070258. 10.1136/bmjopen-2022-070258. 38355188 10.1136/bmjopen-2022-070258 PMC10868307

[23] Rassen J, Bartels D, Schneeweiss S, Patrick A, Murk W. Measuring prevalence and incidence of chronic conditions in claims and electronic health record databases. Clin Epidemiol. 2019. 10.2147/clep.s181242 10.2147/CLEP.S181242 PMC6301730 30588119

[24] Divin N, Given JE, Tan J, Astolfi G, Ballardini E, Barrachina-Bonet L, et al. Antiasthmatic prescriptions in children with and without congenital anomalies: a population-based study. BMJ Open. 2023;13:e068885. 10.1136/bmjopen-2022-068885. 37832979 10.1136/bmjopen-2022-068885 PMC10583066

[25] Schlüter DK, Griffiths R, Adam A, Akbari A, Heaven ML, Paranjothy S, et al. Impact of cystic fibrosis on birthweight: a population-based study of children in Denmark and Wales. Thorax. 2019;74:447–54. 10.1136/thoraxjnl-2018-211706. 30026297 10.1136/thoraxjnl-2018-211706 PMC6484694

[26] Patch C, Charlton J, Roderick P, Gulliford M. Use of antihypertensive medications and mortality of patients with autosomal dominant polycystic kidney disease: a population-based study. Am J Kidney Dis. 2011. 10.1053/j.ajkd.2011.01.023 10.1053/j.ajkd.2011.01.023 21458899

[27] Douglas I, Evans S, Rawlins MD, Smeeth L, Tabrizi SJ, Wexler NS. Juvenile Huntington’s disease: a population-based study using the General Practice Research Database. BMJ Open. 2013;3:e002085. 10.1136/bmjopen-2012-002085. 23558730 10.1136/bmjopen-2012-002085 PMC3641420

[28] Evans SJ, Douglas I, Rawlins MD, Wexler NS, Tabrizi SJ, Smeeth L. Prevalence of adult Huntington’s disease in the UK based on diagnoses recorded in general practice records. J Neurol Neurosurg Psychiatry. 2013;84:1156–60. 10.1136/jnnp-2012-304636. 23482661 10.1136/jnnp-2012-304636 PMC3786631

[29] Wexler NS, Collett L, Wexler AR, Rawlins MD, Tabrizi SJ, Douglas I, et al. Incidence of adult Huntington’s disease in the UK: a UK-based primary care study and a systematic review. BMJ Open. 2016;6:e009070. 10.1136/bmjopen-2015-009070. 26908513 10.1136/bmjopen-2015-009070 PMC4769413

[30] Soriano JB, Lucas SJ, Jones R, Miravitlles M, Carter V, Small I, et al. Trends of testing for and diagnosis of α1-antitrypsin deficiency in the UK: more testing is needed. Eur Respir J. 2018;52:1800360. 10.1183/13993003.00360-2018. 29853490 10.1183/13993003.00360-2018

[31] Wolfson DB, Best AF, Addona V, Wolfson J, Gadalla SM. Benefits of combining prevalent and incident cohorts: an application to myotonic dystrophy. Stat Methods Med Res. 2019;28:3333–45. 10.1177/0962280218804275. 30293502 10.1177/0962280218804275

[32] Hagberg KW, Jick S, Özen G, Du P. Pharmacologically treated anxiety and depression in people diagnosed with von Willebrand disease: matched cohort study. J Blood Med. 2023;14:413–25. 10.2147/JBM.S407993. 37456530 10.2147/JBM.S407993 PMC10349568

[33] Sackley C, Hoppitt TJ, Calvert M, Gill P, Eaton B, Yao G, et al. Huntington’s disease: current epidemiology and pharmacological management in UK primary care. Neuroepidemiology. 2011;37:216–21. 10.1159/000331912. 22133668 10.1159/000331912

[34] Pouwels S, De Boer A, Leufkens HGM, Weber WEJ, Cooper C, Van Onzenoort HAW, et al. Risk of fracture in patients with muscular dystrophies. Osteoporos Int. 2014;25:509–18. 10.1007/s00198-013-2442-2. 23948807 10.1007/s00198-013-2442-2

[35] Evans W, Akyea R, Simms A, Kai J, Qureshi N. Opportunities and challenges for identifying undiagnosed Rare Disease patients through analysis of primary care records: long QT syndrome as a test case. J Commun Genet. 2024. 10.1007/s12687-024-00742-7 10.1007/s12687-024-00742-7 PMC11645366 39405009

[36] Donaldson JW, McKeever TM, Hall IP, Hubbard RB, Fogarty AW. The UK prevalence of hereditary haemorrhagic telangiectasia and its association with sex, socioeconomic status and region of residence: a population-based study. Thorax. 2014;69:161–7. 10.1136/thoraxjnl-2013-203720. 24188926 10.1136/thoraxjnl-2013-203720

[37] Donaldson JW, McKeever TM, Hall IP, Hubbard RB, Fogarty AW. Complications and mortality in hereditary hemorrhagic telangiectasia: a population-based study. Neurology. 2015;84:1886–93. 10.1212/WNL.0000000000001538. 25862798 10.1212/WNL.0000000000001538 PMC4433463

[38] Tresoldi AS, Sumilo D, Perrins M, Toulis KA, Prete A, Reddy N, et al. Increased infection risk in addison’s disease and congenital adrenal hyperplasia. J Clin Endocrinol Metab. 2020;105:418–29. 10.1210/clinem/dgz006. 31532828 10.1210/clinem/dgz006 PMC7046014

[39] Hawley S, Shaw NJ, Delmestri A, Prieto-Alhambra D, Cooper C, Pinedo-Villanueva R, et al. Higher prevalence of non-skeletal comorbidity related to X-linked hypophosphataemia: a UK parallel cohort study using CPRD. Rheumatology. 2021;60:4055–62. 10.1093/rheumatology/keaa859. 33331900 10.1093/rheumatology/keaa859

[40] Kingswood C, Bolton P, Crawford P, Harland C, Johnson SR, Sampson JR, et al. The clinical profile of tuberous sclerosis complex (TSC) in the United Kingdom: a retrospective cohort study in the Clinical Practice Research Datalink (CPRD). Eur J Paediatr Neurol. 2016;20:296–308. 10.1016/j.ejpn.2015.11.011. 26706603 10.1016/j.ejpn.2015.11.011

[41] Kingswood JC, Crawford P, Johnson SR, Sampson JR, Shepherd C, Demuth D, et al. The economic burden of tuberous sclerosis complex in the UK: a retrospective cohort study in the Clinical Practice Research Datalink. J Med Econ. 2016;19:1087–98. 10.1080/13696998.2016.1199432. 27267148 10.1080/13696998.2016.1199432

[42] Kingswood JC, Nasuti P, Patel K, Myland M, Siva V, Gray E. The economic burden of tuberous sclerosis complex in UK patients with renal manifestations: a retrospective cohort study in the Clinical Practice Research Datalink (CPRD). J Med Econ. 2016;19:1116–26. 10.1080/13696998.2016.1202254. 27310569 10.1080/13696998.2016.1202254

[43] Shepherd C, Koepp M, Myland M, Patel K, Miglio C, Siva V, et al. Understanding the health economic burden of patients with tuberous sclerosis complex (TSC) with epilepsy: a retrospective cohort study in the UK Clinical Practice Research Datalink (CPRD). BMJ Open. 2017;7:e015236. 10.1136/bmjopen-2016-015236. 28982809 10.1136/bmjopen-2016-015236 PMC5640029

[44] Wang Y, Pfeiffer R, Alsaggaf R, Meeraus W, Gage J, Anderson L, et al. Risk of skin cancer among patients with myotonic dystrophy type 1 based on primary care physician data from the UK Clinical Practice Research Datalink. Int J Cancer. 2018. 10.1002/ijc.31143 10.1002/ijc.31143 PMC5773358 29114849

[45] Jenkins-Jones S, Parviainen L, Porter J, Withe M, Whitaker M, Holden S, et al. Poor compliance and increased mortality, depression and healthcare costs in patients with congenital adrenal hyperplasia. Eur J Endocrinol. 2018. 10.1530/eje-17-0895 10.1530/EJE-17-0895 29371334

[46] Alsaggaf R, St. George DMM, Zhan M, Pfeiffer RM, Wang Y, Wagner KR, et al. Cancer risk in myotonic dystrophy type I: evidence of a role for disease severity. JNCI Cancer Spectr. 2018;2:pky052. 10.1093/jncics/pky052. 30556050 10.1093/jncics/pky052 PMC6286884

[47] Kuan V, Denaxas S, Izquierdo G, Direk K, Bhatti O, Husain S, et al. A chronological map of 308 physical and mental health conditions from 4 million individuals in the English National Health Service. Lancet Digit Health. 2019. 10.1016/S2589-7500(19)30012-3 10.1016/S2589-7500(19)30012-3 PMC6798263 31650125

[48] Alsaggaf R, Pfeiffer RM, Wang Y, St. George DMM, Zhan M, Wagner KR, et al. Diabetes, metformin and cancer risk in myotonic dystrophy type I. Int J Cancer. 2020;147:785–92. 10.1002/ijc.32801. 31749144 10.1002/ijc.32801 PMC7336337

[49] Hagberg K, Jick S, Du P, Berthoz T, Ozen G, Tzivelekis S. Impact of von Willebrand disease on women’s health outcomes: a matched cohort database study. J Womens Health Larchmt. 2022. 10.1089/jwh.2022.0082 10.1089/jwh.2022.0082 PMC9527044 35960825

[50] Pickrell O, Guelfucci F, Martin M, Holland R, Chin R. Prevalence and healthcare resource utilization of patients with Dravet syndrome: retrospective linkage cohort study. Seizure. 2022;99:159–63. 10.1016/j.seizure.2022.05.018. 35667184 10.1016/j.seizure.2022.05.018

[51] Pimenta JM, Irving M, Cheung M, Mazzeo L, Landis S, Mukherjee S. Higher rates of non-skeletal complications and greater healthcare needs in achondroplasia compared to the general UK population: a matched cohort study using the CPRD database. Orphanet J Rare Dis. 2023;18:211. 10.1186/s13023-023-02811-5. 37491331 10.1186/s13023-023-02811-5 PMC10367327

[52] Wang S, Lau YS, Sutton M, Anderson M, Kypridemos C, Head A, et al. Inequalities in the prevalence recording of 205 chronic conditions recorded in primary and secondary care for 12 million patients in the English National Health Service. BMC Med. 2024;22:570. 10.1186/s12916-024-03767-4. 39623457 10.1186/s12916-024-03767-4 PMC11613489

[53] Alsaggaf R, Pfeiffer R, Pearce E, Greene M, Lochmuller H, Gadalla S. Mortality trends and causes of death in myotonic dystrophy type 1 patients from the UK Clinical Practice Research Datalink. Muscle Nerve. 2024. 10.1002/mus.28308 10.1002/mus.28308 PMC11708454 39679826

[54] Udeze C, Ly N, Ingleby F, Fleming S, Conner S, Howard J, et al. Clinical Burden and Health Care Resource Utilization Associated With Managing Sickle Cell Disease With Recurrent Vaso-occlusive Crises in England. Clin Ther. 2025. 10.1016/j.clinthera.2024.09.023 10.1016/j.clinthera.2024.09.023 39510902

[55] Udeze C, Ly N, Ingleby F, Fleming S, Conner S, Howard J, et al. Clinical Burden and Healthcare Resource Utilization Associated With Managing Transfusion-dependent β-Thalassemia in England. Clin Ther. 2025. 10.1016/j.clinthera.2024.09.024 10.1016/j.clinthera.2024.09.024 39488494

[56] Clift AK, Saatci D, Coupland CAC, Dambha-Miller H, Hippisley-Cox J. Sickle cell disorders and severe COVID-19 outcomes: a cohort study. Ann Intern Med. 2021;174:1483–7. 10.7326/M21-1375. 34338553 10.7326/M21-1375 PMC8343340

[57] Griffiths R, Schlüter DK, Akbari A, Cosgriff R, Tucker D, Taylor-Robinson D. Identifying children with Cystic Fibrosis in population-scale routinely collected data in Wales: a retrospective review. Int J Popul Data Sci. 2020;5. 10.23889/ijpds.v5i1.1346 10.23889/ijpds.v5i1.1346 PMC7898022 33644411

[58] Schlüter DK, Griffiths R, Akbari A, Taylor-Robinson D. Educational achievements of children aged 10–11 years with cystic fibrosis. A data linkage study in Wales. Int J Popul Data Sci. 2022;7. 10.23889/ijpds.v7i1.1725 10.23889/ijpds.v7i1.1725 PMC9284509 35909577

[59] MacRae C, Morales D, Mercer SW, Lone N, Lawson A, Jefferson E, et al. Impact of data source choice on multimorbidity measurement: a comparison study of 2.3 million individuals in the Welsh National Health Service. BMC Med. 2023;21:309. 10.1186/s12916-023-02970-z. 37582755 10.1186/s12916-023-02970-z PMC10426056

[60] Chiovoloni R, Dylag JJ, Alwan NA, Berrington A, Boniface M, Fair N, et al. Cohort profile: creation of the SAIL MELD-B e-cohort (SMC) and SAIL MELD-B children and young adult e-cohort (SMYC) to investigate the lived experience of the ‘burdensomeness’ of multimorbidity. BMJ Open. 2025;15:e087946. 10.1136/bmjopen-2024-087946. 39773797 10.1136/bmjopen-2024-087946 PMC11792564

[61] Furby H, Siadimas A, Rutten-Jacobs L, Rodrigues FB, Wild EJ. Natural history and burden of Huntington’s disease in the UK: a population-based cohort study. Eur J Neurol. 2022;29:2249–57. 10.1111/ene.15385. 35514071 10.1111/ene.15385 PMC9542098

[62] Tyrer F, Morriss R, Kiani R, Gangadharan S, Rutherford M. Mortality disparities and deprivation among people with intellectual disabilities in England: 2000–2019. J Epidemiol Community Health. 2021. 10.1136/jech-2021-216798 10.1136/jech-2021-216798 34244310

[63] Alsaggaf R, St. George DMM, Zhan M, Pfeiffer RM, Wang Y, Anderson LA, et al. Benign tumors in myotonic dystrophy type I target disease-related cancer sites. Ann Clin Transl Neurol. 2019;6:1510–8. 10.1002/acn3.50856. 31402615 10.1002/acn3.50856 PMC6689687

[64] Hawley S, Shaw NJ, Delmestri A, Prieto-Alhambra D, Cooper C, Pinedo-Villanueva R, et al. Prevalence and Mortality of Individuals With X-Linked Hypophosphatemia: A United Kingdom Real-World Data Analysis. J Clin Endocrinol Metab. 2020;105:e871–8. 10.1210/clinem/dgz203. 31730177 10.1210/clinem/dgz203 PMC7025948

[65] Sánchez-Martínez R, Iriarte A, Mora-Luján JM, Patier JL, López-Wolf D, Ojeda A, et al. Current HHT genetic overview in Spain and its phenotypic correlation: data from RiHHTa registry. Orphanet J Rare Dis. 2020;15:138. 10.1186/s13023-020-01422-8. 32503579 10.1186/s13023-020-01422-8 PMC7275435

[66] Rare Disease Research Landscape Steering Group, Bainbridge K. Rare Diseases Research Landscape Project Report. 2023. 10.3310/nihropenres.1115214.1.

[67] IRDiRC State of Play: Rare Diseases Research Initiatives 2019–2021. https://irdirc.org/irdirc-releases-state-of-play-2019-2021/

[68] Thygesen JH, Zhang H, Issa H, Wu J, Hama T, Phiho-Gomes AC, et al. Prevalence and demographics of 331 rare diseases and associated COVID-19-related mortality among 58 million individuals: a nationwide retrospective observational study. Lancet Digit Health. 2025;7:e145–56. 10.1016/S2589-7500(24)00253-X. 39890245 10.1016/S2589-7500(24)00253-X

[69] Fonferko-Shadrach B, Lacey AS, Strafford H, Jones C, Baker M, Powell R, et al. Genetic influences on epilepsy outcomes: a whole-exome sequencing and health care records data linkage study. Epilepsia. 2023;64:3099–108. 10.1111/epi.17766. 37643892 10.1111/epi.17766

[70] Hannah JR, Gordon PA, Galloway J, Rutter M, Peach EJ, Rooney M, et al. Validation of methods to identify people with idiopathic inflammatory myopathies using hospital episode statistics. Rheumatol Adv Pr. 2022;6:rkac102. 10.1093/rap/rkac102. 10.1093/rap/rkac102 PMC9749128 36532317

