# Supplementary material for: Systematic mapping of rare genetic disease studies using UK primary care electronic health records
Source: Eur J Hum Genet. 2026 May 20;34(7):993–1004. doi: 10.1038/s41431-026-02114-w (PMC13341784; doi:10.1038/s41431-026-02114-w)
Supplement: Supplementary file 1 — Supplementary Figures 1-3 [file 41431_2026_2114_MOESM1_ESM.pdf]

European Journal of
Human Genetics

Supplementary Materials

Supplement to: Wright TEB, Slevin H, Magnier S, Carr MJ, Garg S, Webb RT, Ashcroft DM, Banka S. Systematic mapping of rare genetic disease studies using UK primary care electronic health records. *European Journal of Human Genetics*. 2026.

.

Supplementary Figures 1-3 are provided in this Supplementary Materials file:

Supplementary Figure 1. Overview and infrastructure of UK primary care electronic health record research databases.

Supplementary Figure 2. Key strengths of UK primary care electronic health record databases for population-based research.

Supplementary Figure 3. PRISMA diagram for the selection process to identify peer-reviewed publications investigating germline rare genetic diseases using five major UK primary care electronic health record databases.

Supplementary Tables 1-5 are provided in a separate Excel workbook, with each table presented on a dedicated worksheet:

Supplementary Table 1. Characteristics of UK primary care electronic health record databases included in this review.

Supplementary Table 2. Funding of rare genetic disease studies identified in this review using UK primary care electronic health record databases.

Supplementary Table 3. Summary of rare genetic diseases reported in eligible peer-reviewed publications using UK primary care electronic health record databases.

Supplementary Table 4. Condition summary for all Orphanet-classified rare genetic diseases reported in eligible publications using UK primary care electronic health record databases.

Supplementary Table 5. Summary of sample sizes for rare genetic diseases reported in eligible publications using UK primary care electronic health record databases.

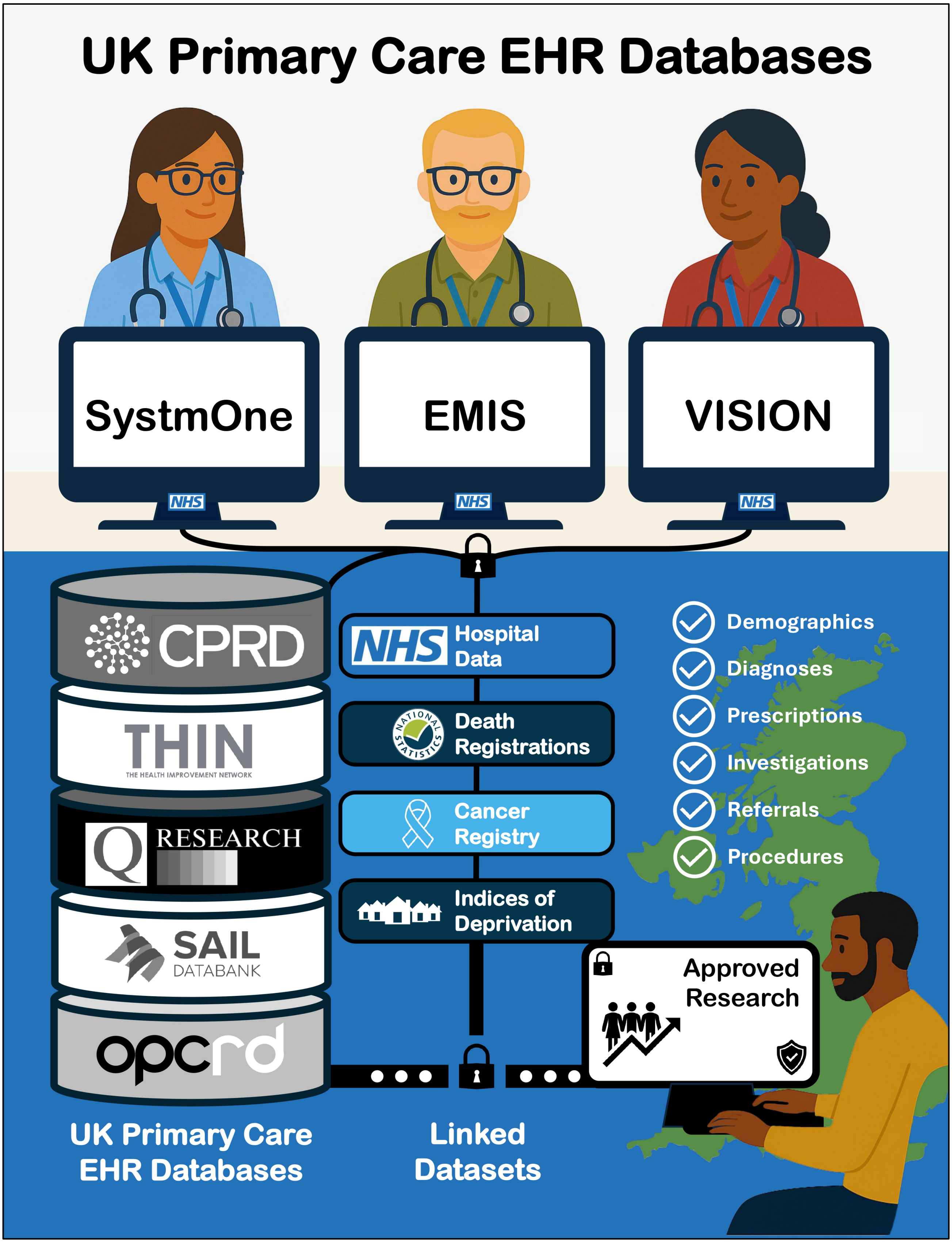

Supplementary Figure 1. Overview and infrastructure of UK primary care electronic health record research databases. *Three NHS primary care clinicians shown using the three main electronic health record systems in UK primary care: SystmOne, EMIS, and Vision. Data from contributing practices are securely transferred to national research databases (illustrated examples: CPRD, THIN, QResearch, SAIL Databank, and OPCRD), which hold routinely collected structured data that can be linked at the individual-level to other national datasets. Approved research is conducted using pseudonymised data within a secure data environment or trusted equivalent.*

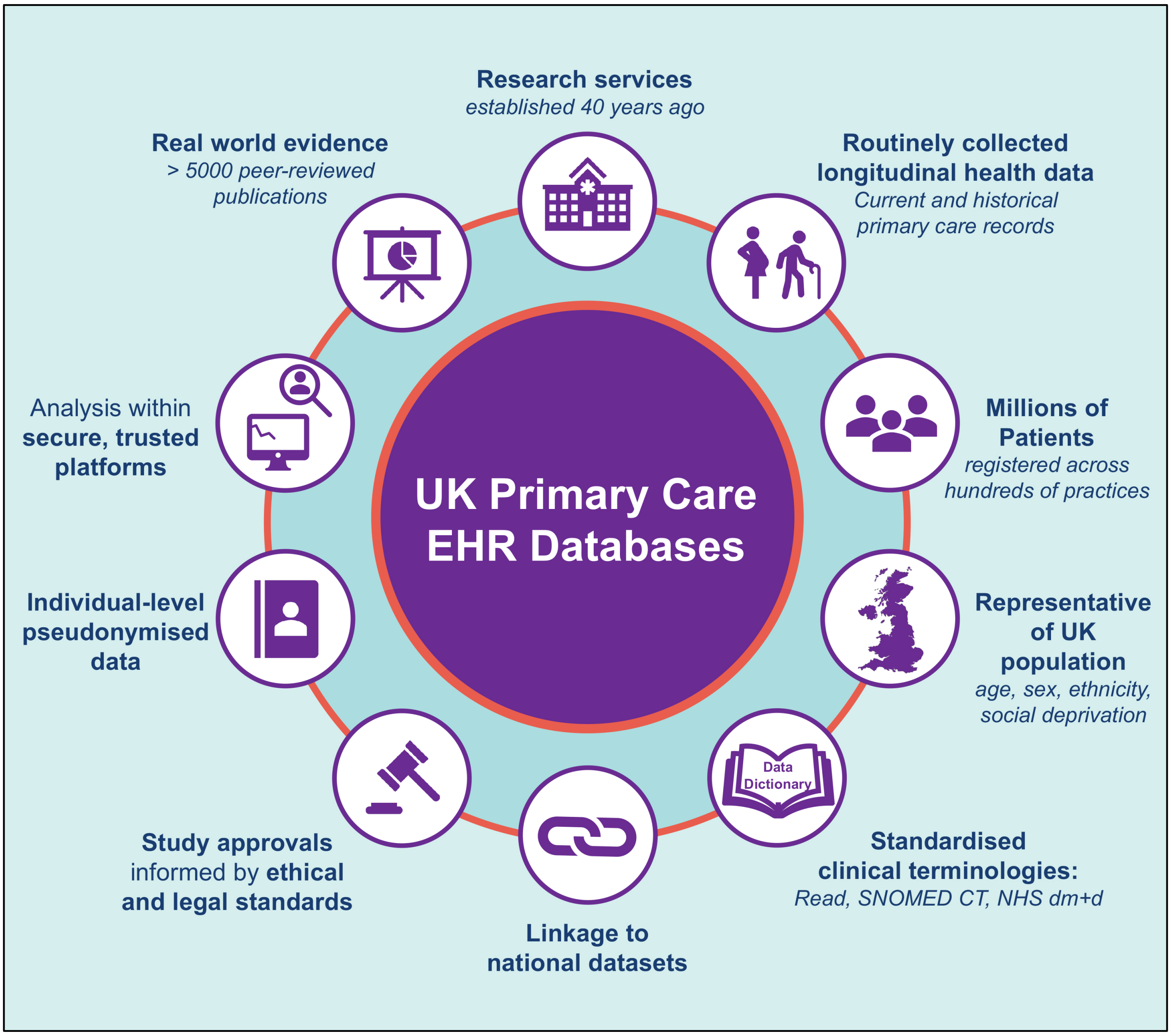

Supplementary Figure 2. Key strengths of UK primary care electronic health record databases for population-based research. *This figure summarises key methodological and infrastructural strengths of UK primary care electronic health record databases for population-based research. These include large and broadly representative populations, routinely collected longitudinal health data coded using standardised terminologies, individual-level linkage to other national datasets, and secure access to pseudonymised data within established governance frameworks. Together, these features have supported a substantial body of real-world evidence across a wide range of research applications.*

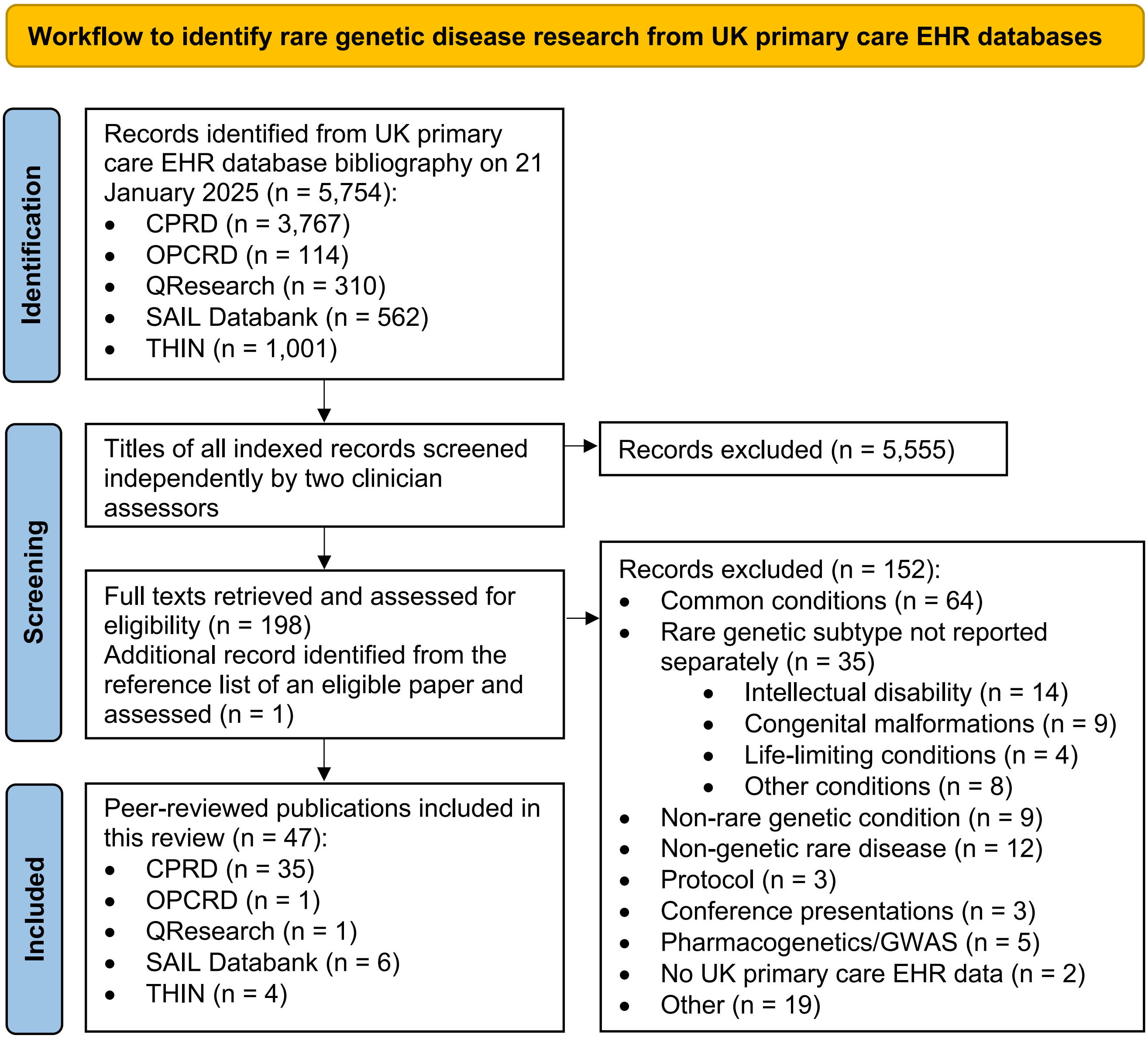

Supplementary Figure 3. PRISMA diagram for the selection process to identify peer-reviewed publications investigating germline rare genetic diseases using five major UK primary care electronic health record databases. *Records were identified from the online bibliographies of five major UK primary care electronic health record research databases (CPRD, OPCRD, QResearch, SAIL Databank, and THIN), accessed on 21 January 2025. All indexed studies were assessed independently by clinician investigators using a two-stage procedure consisting of title screening followed by full text assessment of all potentially relevant articles against predefined eligibility criteria. One additional publication was identified from the reference list of an eligible paper. The diagram summarises the numbers of records screened, assessed for eligibility, excluded, and included in the review. Reasons for excluding studies following full text assessment are shown.*
